# Adaptive Evolutionary Optimization of Deep Learning Architectures for Focused Liver Ultrasound Image Segmentation

**DOI:** 10.3390/diagnostics15020117

**Published:** 2025-01-07

**Authors:** Ali Zifan, Katelyn Zhao, Madilyn Lee, Zihan Peng, Laura J. Roney, Sarayu Pai, Jake T. Weeks, Michael S. Middleton, Ahmed El Kaffas, Jeffrey B. Schwimmer, Claude B. Sirlin

**Affiliations:** 1 Division of Gastroenterology and Hepatology, University of California San Diego, San Diego, CA 92093, USA; k5zhao@ucsd.edu (K.Z.); mal069@ucsd.edu (M.L.); z7peng@ucsd.edu (Z.P.); ljroney@ucsd.edu (L.J.R.); s2pai@ucsd.edu (S.P.); 2 Liver Imaging Group, Department of Radiology, University of California San Diego, San Diego, CA 92093, USA; j1weeks@health.ucsd.edu (J.T.W.); msm.ucsd.edu@gmail.com (M.S.M.); aelkaffas@health.ucsd.edu (A.E.K.); csirlin@health.ucsd.edu (C.B.S.); 3 Department of Pediatrics, Division of Gastroenterology, Hepatology, and Nutrition, University of California San Diego School of Medicine, La Jolla, CA 92093, USA; jschwimmer@health.ucsd.edu; 4 Department of Gastroenterology, Rady Children’s Hospital San Diego, San Diego, CA 92123, USA

**Keywords:** ultrasound liver segmentation, deep learning optimization, evolutionary genetic algorithm

## Abstract

**Background:** Liver ultrasound segmentation is challenging due to low image quality and variability. While deep learning (DL) models have been widely applied for medical segmentation, generic pre-configured models may not meet the specific requirements for targeted areas in liver ultrasound. Quantitative ultrasound (QUS) is emerging as a promising tool for liver fat measurement; however, accurately segmenting regions of interest within liver ultrasound images remains a challenge. **Methods:** We introduce a generalizable framework using an adaptive evolutionary genetic algorithm to optimize deep learning models, specifically U-Net, for focused liver segmentation. The algorithm simultaneously adjusts the depth (number of layers) and width (neurons per layer) of the network, dropout, and skip connections. Various architecture configurations are evaluated based on segmentation performance to find the optimal model for liver ultrasound images. **Results:** The model with a depth of 4 and filter sizes of [16, 64, 128, 256] achieved the highest mean adjusted Dice score of 0.921, outperforming the other configurations, using three-fold cross-validation with early stoppage. **Conclusions:** Adaptive evolutionary optimization enhances the deep learning architecture for liver ultrasound segmentation. Future work may extend this optimization to other imaging modalities and deep learning architectures.

## 1. Introduction

Metabolic dysfunction-associated steatotic liver disease (MASLD) affects approximately 10% of children and, if left untreated, may progress to its more advanced form, metabolic dysfunction-associated steatohepatitis (MASH), and to long-term complications such as diabetes, cardiovascular disease, cirrhosis, and liver cancer [1,2,3,4,5]. Magnetic resonance imaging proton-density fat fraction (MRI-PDFF) is widely recognized as the non-invasive reference standard to assess hepatic steatosis, the hallmark feature of MASLD [6,7]; however, its availability is limited due to its infrastructure requirements, and costs [8], especially in resource-limited regions worldwide. In contrast, ultrasound (US) is safe, non-invasive, more affordable, and more widely available, but may underdiagnose mild steatosis due to operator and machine dependence, and reliance on reader assessment [9,10].

Appreciation of the limitations of US has sparked growing interest in developing quantitative ultrasound (QUS) as an alternative technology to assess possible MASLD [11,12]. Although QUS has shown promise to be more accurate and precise than US, additional research is needed to fully validate QUS [13]. In one approach, the liver boundaries are determined using manual US liver segmentation, which is time-consuming and labor-intensive, and then, hepatic steatosis and perhaps other histologic features of MASLD are assessed using the QUS methodology. An important step to improve QUS liver segmentation is to automate liver boundary delineation, as this is currently performed manually by radiologists.

Deep learning techniques have proven efficacious in segmenting liver MRI and CT [14,15,16,17,18,19,20,21,22,23], but not yet US images [1,24,25,26,27,28,29,30,31,32]. Moreover, existing liver US approaches are limited and have seen only limited mainstream application due to the lack of sufficient data or reproducibility [33]. The aim of this study is to address these challenges by advancing toward a fully automated targeted-field-of-interest liver US segmentation by using genetic evolutionary algorithms to determine the optimal neural network architecture.

## 2. Materials and Methods

To achieve automated and robust targeted-region-of-interest ultrasound (US) liver segmentation, we introduce a new method to optimize an established encoder–decoder model such as the U-Net [34] deep learning (DL) architecture using evolutionary algorithms (EAs), though it should be noted that any other model could be used, as the goal is architectural optimization. Evolutionary algorithms are optimization techniques inspired by natural selection processes that iteratively refine solutions by simulating evolution. EAs provide a powerful framework for exploring and refining network architecture; they naturally lend themselves to parallel processing and help speed up the search process. Genetic algorithms (GAs), a subset of EAs, use mechanisms like selection, crossover, and mutation to evolve solutions over generations. GAs are particularly useful when computation times for individual evaluations are long, making them well suited to solving problems that require global search, robustness, and flexibility [35]. Natural selection is a process in biology where organisms better adapted to their environment tend to survive and reproduce, passing on advantageous traits to future generations. By simulating natural selection, evolutionary algorithms (EAs) iteratively evolve deep learning (DL) architectures to enhance liver segmentation performance. Mutation is a process where random changes are introduced to the architecture, allowing new variations that may improve performance. Crossover combines features from two parent architectures to create offspring that could potentially inherit strengths from both. Migration involves transferring solutions or information between populations, encouraging diversity in the search space. Selection is the process of choosing the best-performing architectures to pass their features to the next generation. Through these mechanisms, candidate architectures are generated and evaluated based on segmentation accuracy, allowing U-Net models to adapt and optimize for liver ultrasound segmentation without manual tuning.

### 2.1. Experimental Setup

Ultrasound data were acquired from 30 children (mean age: 13 ± 2.4 years). Among the participants, 12 (40%) had a clinical diagnosis of MASLD, 17 (56.7%) were at risk for MASLD, and 1 (3.3%) had neither. We used the C1-6 curved array probe of a GE Logiq E10 system (GE HealthCare, Chicago, IL, USA). The study was approved by the UCSD IRB. Parents gave written consent and participants gave written assent. Transverse B-mode images of the right lobe of the liver were acquired through an intercostal window in both the fundamental and harmonic modes by one of two study-trained registered diagnostic medical sonographers. For each child, the sonographer selected the settings that optimized liver visualization in their judgment. These settings included gain, time-gain compensation, depth, and transmit center frequency (3.0 MHz or 4.0 MHz for fundamental mode, 3.0 MHz or 4.5 MHz for harmonic mode). Transverse scanning was chosen to standardize the imaging protocol and ensure consistent visualization of liver boundaries, which are critical for evaluating segmentation performance. While we acknowledge its limitations in visualizing deeper liver segments (e.g., segments 6 and 7), this approach minimizes variability introduced by operator-dependent factors, such as probe angle and patient positioning. Future work will explore integrating oblique or intercostal scanning views to address challenges related to sound refraction and ultrasound attenuation through the abdominal rectus muscle.

Liver boundaries on each B-mode image were segmented manually by a trained image analyst under the supervision of a radiologist using the ITK-SNAP segmentation tool. The field of interest was drawn to capture as much liver parenchyma as possible while avoiding liver edges, shadows, dropout, and other artifacts. No effort was made to avoid blood vessels in the field of interest, as also our previous preliminary data indicated that vessel removal did not affect the results.

The B-mode images were subsequently loaded on a Dell Precision T7910, Dual Intel Xeon Processor E5-2687W v4, NVIDIA Quadro M6000 24 GB, 256 GB RAM. OpenAI’s generative AI tools were used to assist with language editing and grammar correction. The tools were employed exclusively for refining the text and did not contribute to the conceptualization, data analysis, or interpretation of the study results. We used Python 3.8 (Python Software Foundation, https://www.python.org), and Keras [36] for our EA implementation, which also facilitated multiprocessing, enabling the simultaneous training of multiple genomes for the liver segmentation task.

### 2.2. Evolutionary Genomic Optimization

**Figure 1:**
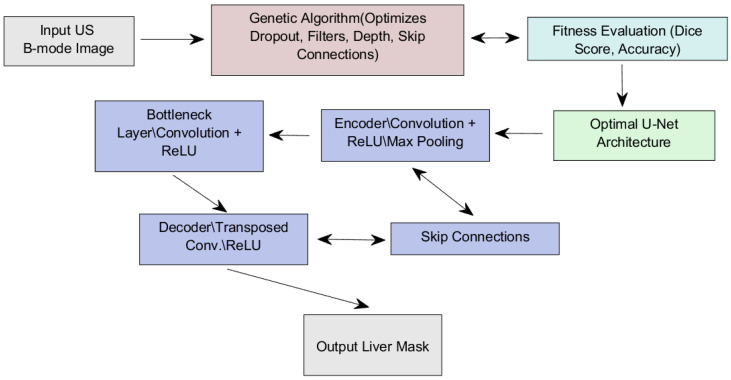
A flow diagram of the proposed genomic optimization of the U-Net model.

We employed a multi-population evolutionary approach to enhance the training of our predictive U-Net model (see Figure 1). This method involved simultaneously training across multiple genomic subpopulations, allowing us to leverage their unique characteristics and improve the model’s robustness and generalizability.

We used parallel genomic training sessions with varying depths (see Table 1) to capture a broader spectrum of features and interactions. By systematically varying the model’s depth, dropout rate, and skip connections (set to True or False), we were able to explore different levels of complexity in the abdominal ultrasound images, ensuring that both shallow and deep representations of the genomic information were effectively learned. Moreover, we integrated migration techniques to facilitate the transfer of knowledge between subpopulations. This enabled the model to retain learned representations from shallower depths and apply them to deeper models, enhancing its ability to recognize and classify patterns across diverse genomic landscapes. The novelty of this approach is the combined effect of these methodologies, which allows us to create a flexible and adaptable deep learning model architecture that could effectively interpret the complex relationships within the liver US genomic data.

- **Genome Representation**: We represent each genome as a dictionary containing the following hyperparameters:
  ▪ **Dropout Rate**: $p_{d}$, where $p_{d}\in \left[0,\;0.5\right]$.
  ▪ **Filter Sizes**: a list of integers representing the number of filters in each layer (see Table 1), $F=\left[f_{1},f_{2},f_{3},f_{4}\right]$.
  ▪ **Depth**: *d*, representing the number of layers, where *d* ∈ [2, 5].
  ▪ **Use Skip Connections**: a Boolean flag $u_{s}$, indicating whether skip connections are included.
- **Fitness Function:** the fitness of each U-Net genome is evaluated based on the Dice coefficient:

(1) $$Dice\left(y_{true},y_{pred}\right)=\frac{2\cdot \left|y_{true}\cap y_{pred}\right|}{\left|y_{true}\right|+\left|y_{pred}\right|}+\epsilon$$

where ϵ is a small constant to prevent division by zero. Additionally, we define the ***average fitness*** across the population:

(2) $$Avg\;Fitness=1/N\sum_{i=1}^{N}{Fitness}_{i}$$

where *N* is the population size.

- **Selection:** The population is sorted based on fitness scores, and the top half of the genomes is retained for the next generation. The selected genome can be represented as

(3) $$Winner\;Genome=argmax\left({Fitness}_{i}\right),\;\;\;\;\;i\in [1,N]$$

- **Crossover:** Two parent genomes are randomly selected to produce offspring through the following rules:
  ▪ The dropout rate and depth are averaged:
    (4) $$p_{d,child}=\frac{p_{d,parent1}+p_{d,parent2}}{2}\;d_{child}=round\left(\frac{p_{d,parent1}+p_{d,parent2}}{2}\right)$$
  ▪ The filter sizes are averaged and rounded to the nearest integer:
    (5) $$f_{i,child}=round\left(\frac{f_{i,parent1}+f_{i,parent2}}{2}\right),\;i\in [1,N]$$
  ▪ The skip connection flag is randomly selected from the parents.
- **Migration:** Facilitates the transfer of knowledge between subpopulations:
  ▪ After every few generations, a certain percentage of genomes are migrated between subpopulations. This can be represented as
    (6) $$p_{d,migrated}=\left(1-migration\;rate\right)\cdot p_{d,original}+migration\;rate\cdot p_{d,source}$$
  ▪ This influences the fitness evaluation and crossover processes.
- **Mutation**: Random mutations are applied to introduce variability:
  ▪ With a probability of 10%, the dropout rate is perturbed:
    (7) $$p_{d,mutated}=p_{d,child}+∆p,\;\;\;\;\;\;\;∆p\sim u(-0.05,0.05)$$
  ▪ With a probability of 10%, each filter size is adjusted by ±8 filters, ensuring the values stay within the valid range:
    (8) $$f_{i,mutated}=clip\left(f_{i,child}+∆f,{min}_{f},{max}_{f}\right),\;\;\;\;∆f\sim u(-8,8)$$

In our setting, we select the top-performing genome from each subpopulation (depth) based on fitness (Dice) scores, transferring its dropout rate and skip connection configuration to other subpopulations. Next, when a genome migrates, new filter sizes are generated to align with the target depth’s number of layers, ensuring compatibility. Subsequently, each depth retains only the top 3 genomes after migration, maintaining a focused search within each subpopulation while allowing beneficial traits to spread across different depths.

- **Boundary Constraints:** To maintain valid parameter ranges, we apply clipping for the filter sizes and dropout rates:
  (9) $$f_{i,constrained}=clip\left(f_{i},f_{min},f_{max}\right)\;p_{d,constrained}=clip\left(p_{d},0,0.5\right)$$
- **Depth Penalization:** We also penalize deeper networks to prevent overfitting and manage the trade-off between model complexity and performance (avoiding extra training parameters):
  (10) Adjusted Dice score=Best Dice·1d·p,
  where *d: depth* and *p: penalty factor.*
- **Convergence Criteria:** We run the algorithm for a predefined number of generations (e.g., 30 epochs), *gen*, or until the change in average fitness across generations is below a threshold $\epsilon_{c}$:
  (11) $$Convergence=if\;\left|{Avg\;Fitness}_{gen}-{Avg\;Fintess}_{gen-1}\right|<\epsilon_{c}$$
- **Training Process**

The best genome identified by the GA is used to configure the U-Net model. The model is trained on the preprocessed dataset with the following loss function:

(12) $$Loss=-(y_{true}\log\left(y_{pred}\right)+\left(1-y_{true}\right)\log\left(1-y_{pred}\right))$$

Next, the model is optimized using an Adam optimizer with a learning rate of α set at 1 × 10^−4^. The performance of the model is evaluated using the Dice coefficient as the primary metric.

## 3. Results

**Figure 2:**
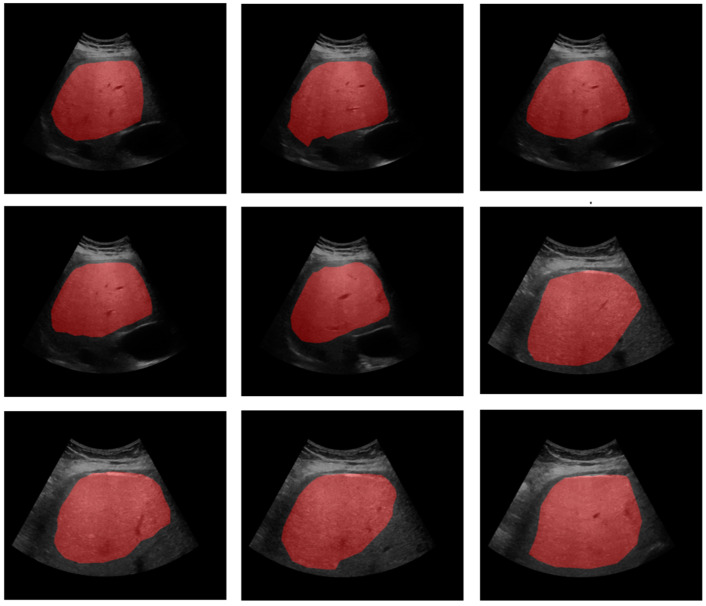
Nine samples of different subjects with superimposed ground truth segmentation.

In our optimization approach, the first step involved genome optimization to identify the best architecture, as described previously. This involved fine-tuning key parameters, including filter sizes, depth, skip connections, and dropout rates. To expedite the identification of an initial winner, we employed a smaller number of epochs (30) during this phase, utilizing an 80-20 train–test split for model evaluation on 627 analyst-labeled 256-by-256 liver ultrasound images (see Figure 2). The optimization was facilitated through a combination of crossover, mutation, and migration techniques, along with penalizing larger depths using a factor of 0.1, thereby encouraging the exploration of more efficient architectures. The second step utilized the optimized architecture identified in the first phase, increasing the number of sampling epochs to 300 (with early stoppage) to achieve further refinement of the winning architecture. By passing the best-performing model from one generation to the next (10 generations), we introduced additional modifications through mutation and crossover, which led to the evolution of increasingly effective models. The proposed segmentation framework prioritizes time and cost efficiency: the evolutionary optimization process converged within 48 GPU hours, while the optimized model processes each image in under 0.1 s on a standard GPU.

**Figure 3:**
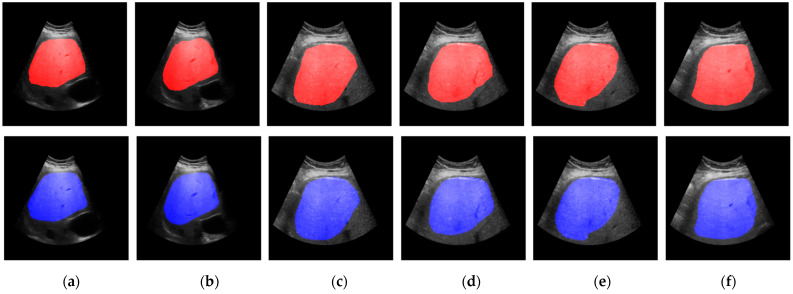
The segmentation results showing the original images with ground truth segmentation overlaid in red at the top and predicted segmentation in blue at the bottom for three samples: (**a**–**f**).

The phase 1 (i.e., multi-population genomic optimization) results revealed that the model with a depth of 4 and filter sizes of [16, 64, 128, 256] emerged as the top performer, achieving an adjusted Dice score of 0.859 on 627 images with a size of 256 by 256. This score not only outperformed the other configurations tested but also ranked as the best architecture among all 10 generations (see Table 2). In comparison, the depth 3 model with filter sizes of [16, 32, 128] achieved an adjusted Dice score of 0.685. The depth 5 model, configured with filter sizes of [16, 32, 64, 128, 256], scored 0.828, while the depth 6 model, utilizing filter sizes of [32, 64, 128, 256, 512, 1024], yielded an adjusted score of 0.826. An interesting collateral of these results is that increasing depth and complexity does not inherently lead to improved performance, as indicated by the lower scores of the depth 5 and depth 6 models compared to the depth 4 configuration. Naturally, as generations progressed, the architectures exhibited noticeable improvements in performance, underscoring the effectiveness of the proposed optimization strategy. The proposed evolutionary pipeline successfully identified the depth 4 architecture with filter sizes of [16, 64, 128, 256] and a dropout rate of 0.1464 (with skip connections) as the optimal choice for the segmentation of the US liver dataset. This configuration not only demonstrated superior performance but also consistently ranked among the top architectures across all generations. Finally, the optimal genome was used to train the U-Net model with early stopping (max 300 epochs) to obtain the final model. This resulted in a mean Dice score of 0.92 with a standard deviation of 0.00124 across three folds. See Table 2 and Figure 3 for the sample results of the prediction model applied on six random US samples in the dataset. Representative examples of segmented liver boundaries were overlaid on the original ultrasound images to illustrate the model’s performance. These visualizations highlight the method’s ability to accurately delineate liver parenchyma despite challenges in low-quality ultrasound data.

## 4. Discussion

We employed evolutionary algorithms to optimize the architecture of a deep learning model, specifically a U-Net, for segmenting the liver boundary in abdominal ultrasound images from children. The proposed segmentation method offers several critical contributions to clinical applications: (A) Liver Fat Quantification: By enabling accurate delineation of liver parenchyma, this method ensures precise measurements of QUS parameters such as the attenuation coefficient and backscatter coefficient. These parameters are foundational for diagnosing conditions like MASLD or liver fibrosis. (B) Early Diagnosis of Liver Cirrhosis: The proposed segmentation allows for the automated extraction of liver texture and morphological features, which serve as biomarkers for cirrhosis detection. (C) Enhanced Workflow Consistency: By automating ROI selection, our method reduces operator dependency, improving the reproducibility and reliability of QUS analysis.

Moreover, by iteratively refining parameters such as dropout rates, filter sizes, depth, and skip connections, we demonstrated that this approach could lead to optimized architectures. While our study focused on the U-Net architecture, the proposed optimization methodology can be readily applied to any network architecture which has an encoder–decoder design, offering a flexible and effective solution for improving segmentation outcomes across various domains. Our results indicate that evolutionary optimization has the potential to substantially boost the accuracy and robustness of deep learning models in ultrasound liver segmentation. Moreover, by leveraging open-source tools and eliminating the need for proprietary software, implementation costs were minimized. Furthermore, the automated segmentation reduces reliance on manual annotations, saves valuable time for clinicians, and ensures the framework’s scalability and feasibility for diverse clinical and operational settings.

The main goal of our proposed method was to optimize deep learning architectures to maximize segmentation quality and accuracy, particularly for challenging low-quality ultrasound images. While the current architecture achieves competitive processing speed, further optimizations are possible. For example, one could adopt lightweight design strategies such as depthwise separable convolutions, which split standard convolutions into depthwise and pointwise operations. This could reduce the computational burden while retaining performance. Or we could also explore techniques like group convolutions, channel shuffling, or even attention mechanisms to further improve efficiency by reducing redundancy, with an augmented focus on computation. While such adaptations are beyond the scope of this study, they represent directions for enhancing speed without compromising segmentation quality, particularly in real-time or resource-constrained clinical settings.

However, our study is not without limitations. Other limitations include using a single transverse image, focusing on children only, employing one transducer (GE LOGIQ E10) only, and utilizing an intercostal view only. One notable constraint is restricting the filter sizes to powers of two (as is commonly used, as they align better with computer architecture for optimal memory management and processing efficiency). Although this choice enhances computational performance and simplifies batch processing, leading to a more structured and scalable network design, it inherently constrains the search space and the random evolutionary nature of the method, potentially overlooking filter sizes that are not powers of two. In a pure evolutionary setting, filter sizes can vary widely, and architectural configurations need not necessarily adhere to a funnel shape or a strictly monotonic increase or decrease. However, filter sizes were restricted to powers of two to optimize computational efficiency and compatibility with GPU memory alignment. Additional experiments with unrestricted filter sizes demonstrated negligible performance improvements (<1% Dice score increase) while significantly increasing computational costs. This practical design choice ensures an optimal balance between performance and efficiency. Therefore, our future studies could benefit from a more expansive exploration of filter sizes, allowing for a more diverse set of architectures that may yield superior performance.

Furthermore, the mutation strategy employed in our genetic algorithm could also be further enhanced. While the primary focus of our genomic optimization was on optimizing filter size and depth, dropout and skip connections, alternative mutation strategies might lead to even better outcomes. However, exploring different mutation mechanisms was outside the scope of our paper, but remains an intriguing avenue for future research. Another drawback of using genetic algorithms is the considerable training time they entail, particularly given the computational complexity involved in evaluating multiple generations of architectures. To address this challenge, we leveraged the multiprocessing capabilities provided by Python’s multiprocessing module, specifically using the ‘Pool’, ‘Manager’, and ‘Lock’ classes, which allowed us to simultaneously train different genomes, significantly reducing the overall computational burden and expediting the optimization process.

Finally, one could explore hybrid strategies. For example, while we applied genetic optimization for optimizing all genome features at once, an alternative strategy could be to integrate differential evolution [37] techniques for optimizing hyperparameters such as dropout rates and learning rates while confining genetic optimization specifically for only filter and depth optimization. This hybrid approach could offer a more comprehensive optimization framework, allowing for finer control over various aspects of the network architecture and training process, also including the learning rate itself as an additional search parameter, allowing us to achieve a balance between faster convergence and model stability, enhancing the overall training efficiency. Reproducibility and generalizability remain critical challenges in medical imaging studies. Factors such as vendor variability, acquisition protocols, and patient demographics influence reproducibility. Our future work will address these challenges by incorporating data from multiple ultrasound vendors, standardizing acquisition protocols, and including diverse patient populations with liver pathologies. Importantly, the proposed methodology is not tied to specific devices, making it adaptable to various clinical and research settings.

## 5. Conclusions

In summary, our results suggest that genomic optimization using evolutionary algorithms is a highly promising avenue for optimizing deep learning architectures in field-of-interest liver ultrasound segmentation. This process allows the algorithmic models to adapt to the specific needs of the segmentations being performed. By utilizing parallelization, multiple architectural representations can be trained simultaneously on any encoder–decoder architecture, deriving the best-performing hyperparameters for a specific segmentation task. Though we applied our methods to liver ultrasound segmentation, this approach is generalizable and can be readily applied to the segmentation of other organs across different imaging modalities.

## Figures and Tables

**Table 1:** Filter sizes explored during the evolutionary optimization of the U-Net architecture.

Depth
3
[8, 16, 128], [8, 32, 128], [16, 32, 128]
[8, 64, 256], [64, 128, 256], [16, 32, 64], [32, 64, 128]
4
[16, 32, 64, 128], [64, 128, 256, 512], [8, 32, 128, 256]
[8, 64, 128, 512], [16, 64, 128, 256], [32, 64, 128, 256]
5
[16, 32, 64, 128, 256], [32, 64, 128, 256, 512], [8, 32, 128, 256, 512],
[8, 64, 256, 512, 1024], [64, 128, 256, 512, 1024]
6
[32, 64, 128, 256, 512, 1024], [8, 16, 64, 128, 256, 512], [16, 32, 64, 128, 256, 512]
[8, 32, 128, 256, 512, 1024], [16, 32, 128, 256, 512, 1024]

**Table 2:** U-Net architectures ^1^ at varying depths across ten generations of evolutionary optimization.

Generation	Depth and Filter Sizes/Adjusted Scores
Gen 1	3: [8, 64, 256] (0.66874), 4: [64, 128, 256, 512] (0.85492), 5: [16, 32, 64, 128, 256] (0.86036), 6: [32, 64, 128, 256, 512, 1024] (0.85848)
Gen 2	3: [64, 128, 256] (0.49106), 4: [64, 128, 256, 512] (0.79684), 5: [16, 32, 64, 128, 256] (0.79872), 6: [32, 64, 128, 256, 512, 1024] (0.87574)
Gen 3	3: [8, 64, 256] (0.75624), 4: [64, 128, 256, 512] (0.86317), 5: [16, 32, 64, 128, 256] (0.84136), 6: [32, 64, 128, 256, 512, 1024] (0.77848)
Gen 4	3: [8, 64, 256] (0.60629), 4: [64, 128, 256, 512] (0.88488), 5: [16, 32, 64, 128, 256] (0.77838), 6: [32, 64, 128, 256, 512, 1024] (0.79681)
Gen 5	3: [64, 128, 256] (0.67891), 4: [64, 128, 256, 512] (0.85566), 5: [16, 32, 64, 128, 256] (0.79540), 6: [32, 64, 128, 256, 512, 1024] (0.78476)
Gen 6	3: [8, 64, 256] (0.85739), 4: [16, 64, 128, 256] (0.76498), 5: [16, 32, 64, 128, 256] (0.83489), 6: [32, 64, 128, 256, 512, 1024] (0.83680)
Gen 7	3: [8, 64, 256] (0.63057), 4: [16, 64, 128, 256] (0.83652), 5: [16, 32, 64, 128, 256] (0.83393), 6: [8, 32, 128, 256, 512, 1024] (0.76690)
Gen 8	3: [8, 64, 256] (0.76110), 4: [16, 64, 128, 256] (0.80789), 5: [16, 32, 64, 128, 256] (0.73106), 6: [32, 64, 128, 256, 512, 1024] (0.84866)
Gen 9	3: [16, 32, 128] (0.43480), 4: [16, 64, 128, 256] (0.81051), 5: [64, 128, 256, 512, 1024] (0.81905), 6: [8, 32, 128, 256, 512, 1024] (0.78400)
Gen 10	3: [16, 32, 128] (0.68542), 4: [16, 64, 128, 256] (0.85939), 5: [16, 32, 64, 128, 256] (0.82776), 6: [32, 64, 128, 256, 512, 1024] (0.82564)

^1^ The results from the optimization process in the study on U-Net architecture for US liver segmentation. The adjusted scores represent the performance metric for each model configuration, with higher scores indicating better segmentation accuracy. The filter sizes are specified in brackets, and the adjusted Dice scores in parenthesis.

## Data Availability

The data presented in this study are available on request from the corresponding author. The data are not publicly available due to IRB.
